# Enhancing evidence informed policymaking in complex health systems: lessons from multi-site collaborative approaches

**DOI:** 10.1186/s12961-016-0089-0

**Published:** 2016-03-17

**Authors:** Etienne V. Langlois, Victor Becerril Montekio, Taryn Young, Kayla Song, Jacqueline Alcalde-Rabanal, Nhan Tran

**Affiliations:** Alliance for Health Policy and Systems Research, World Health Organization, 20 avenue Appia, 1211 Geneva, Switzerland; Center for Health Systems Research, National Institute of Public Health - Instituto Nacional de Salud Pública, Av. Universidad No. 655 Colonia Santa María Ahuacatitlán Cerrada Los Pinos y Caminera C.P., 62100 Cuernavaca, Morelos Mexico; Centre for Evidence-based Health Care, Department of Interdisciplinary Health Sciences, Faculty of Medicine and Health Sciences, Stellenbosch University, PO Box 241, Cape Town, 8000 South Africa; Cochrane South Africa, South African Medical Research Council, Cape Town, South Africa; Health Systems Performance Research Network, Institute of Health Policy, Management and Evaluation, University of Toronto, 155 College Street, Suite 425, Toronto, Ontario M5T 3M6 Canada

**Keywords:** Capacity strengthening, Communities of practice, Complex health system, Decision making, Embedded research, Evidence-informed policy, Health policy, Health systems research, Low- and middle-income countries, Policymaking, Systematic reviews, Use of evidence

## Abstract

**Background:**

There is an increasing interest worldwide to ensure evidence-informed health policymaking as a means to improve health systems performance. There is a need to engage policymakers in collaborative approaches to generate and use knowledge in real world settings. To address this gap, we implemented two interventions based on iterative exchanges between researchers and policymakers/implementers. This article aims to reflect on the implementation and impact of these multi-site evidence-to-policy approaches implemented in low-resource settings.

**Methods:**

The first approach was implemented in Mexico and Nicaragua and focused on implementation research facilitated by communities of practice (CoP) among maternal health stakeholders. We conducted a process evaluation of the CoPs and assessed the professionals’ abilities to acquire, analyse, adapt and apply research. The second approach, called the Policy BUilding Demand for evidence in Decision making through Interaction and Enhancing Skills (Policy BUDDIES), was implemented in South Africa and Cameroon. The intervention put forth a ‘buddying’ process to enhance demand and use of systematic reviews by sub-national policymakers. The Policy BUDDIES initiative was assessed using a mixed-methods realist evaluation design.

**Results:**

In Mexico, the implementation research supported by CoPs triggered monitoring by local health organizations of the quality of maternal healthcare programs. Health programme personnel involved in CoPs in Mexico and Nicaragua reported improved capacities to identify and use evidence in solving implementation problems. In South Africa, Policy BUDDIES informed a policy framework for medication adherence for chronic diseases, including both HIV and non-communicable diseases. Policymakers engaged in the buddying process reported an enhanced recognition of the value of research, and greater demand for policy-relevant knowledge.

**Conclusions:**

The collaborative evidence-to-policy approaches underline the importance of iterations and continuity in the engagement of researchers and policymakers/programme managers, in order to account for swift evolutions in health policy planning and implementation. In developing and supporting evidence-to-policy interventions, due consideration should be given to fit-for-purpose approaches, as different needs in policymaking cycles require adapted processes and knowledge. Greater consideration should be provided to approaches embedding the use of research in real-world policymaking, better suited to the complex adaptive nature of health systems.

**Electronic supplementary material:**

The online version of this article (doi:10.1186/s12961-016-0089-0) contains supplementary material, which is available to authorized users.

## Background

As the global health community is pushing for ambitious goals of universal health coverage and health equity in the post-2015 development era, there is an increasing interest worldwide to ensure evidence-informed health policymaking as a means to improve health systems performance [1]. Use of evidence in health systems strengthening and policymaking plays an essential role in improving service delivery [2, 3]. Evidence uptake to support effective and efficient health systems interventions is paramount in the specific contexts of resource scarcity and high burdens of disease in low- and middle-income countries (LMICs) [4, 5]. The use of research findings is also fundamental to enhance the responsiveness of health systems [5]. Numerous global health initiatives have promoted knowledge application into policy [6], yet the use of evidence by policymakers to inform programmes aligned with population needs still remains an important public health challenge.

In recent years, various strategies were developed and tested to bridge the gap between research and policy, largely focusing on knowledge translation (KT) [7]. A number of strategies have promoted policy dialogues [8], capacity development of researchers to communicate their research results to policymakers [9], knowledge brokerage mechanisms [10], as well as media-based strategies [11]. Although some strategies have yielded positive results, conventional KT approaches have been criticised for failing to adequately take into account the complexity of health policymaking processes and its inherent power dynamics [12, 13]. In addition, KT models often lack specificity on contexts pertaining to LMICs [14]. Furthermore, there is a need to explicitly consider health equity issues in knowledge management approaches to support policymaking, especially in LMIC settings where health inequities are considerable [15]. The prevailing practices thus seem necessary, but not sufficient, to support evidence-informed policy and numerous voices have called for more effective and innovative mechanisms to bridge the divide between knowledge generation and uptake [16, 17]. In particular, there is a need to engage policymakers at all levels in the production and use of knowledge.

Previous research has shown that interactions between researchers and policymakers, timely access to quality evidence, and skills-building with policymakers increase the prospect of research findings being used in policy formulation [18, 19]. There is a growing interest in researcher-policymaker exchanges and engagement, which seem better suited for the complex nature of policymaking processes in comparison to static generalizable research [20]. Consequently, there is a need for collaborative approaches catalysing the integration of health research into policy and program processes.

The WHO Strategy on Health Policy and Systems Research entitled ‘Changing Mindsets’ stressed that research should be demand-driven and not viewed only as an activity, “*wherein researchers pursue an area of interest and expect decision makers to readily alter policy in response to the results*” [21]. In order to generate more demand for research evidence, there is a need to strengthen the value attributed to research and to build a strong culture of evidence-informed policymaking. Furthermore, a managerial and organizational culture has been described as crucial to the development of a climate conducive to the adoption of science and to the greater ownership of evidence-to-policy processes [22]. Recognising the importance of research to support health system strengthening, it has been argued that evidence uptake mechanisms and pathways should be a priority for health systems research on a global scale [23, 24].

It is against this backdrop that the Alliance for Health Policy and Systems Research, a partnership housed within WHO, has developed a programme of work entitled ‘Leadership Development for Enhanced Decision Making’, with the aim of identifying innovative approaches to building skills and competencies for policymakers at all levels within the health system*.* This programme specifically focused on strengthening the capacities of health system actors to access, evaluate and use research evidence to support complex policymaking processes. The first approach supported under this scheme was implemented in Mexico and Nicaragua, and the second one in South Africa and Cameroon. This article aims to reflect on lessons from the implementation and impact of the multi-site evidence-to-policy approaches implemented in low-resource settings. We identify commonalities in how they engaged stakeholders and fostered collaboration between researchers and policymakers or implementers. We draw valuable lessons for supporting evidence-informed policymaking in complex health systems in LMICs.

## Methods

In 2012, the Alliance for Health Policy and Systems Research issued an open call for proposals with a three-fold objective: (1) promoting research uptake in health program and policy implementation to address Millennium Development Goals 4 (Reduce Child Mortality), 5 (Improve Maternal Health) and 6 (Combat HIV/AIDS, Malaria and other diseases); (2) strengthening the capacity of policymakers to communicate their need for evidence, and access and use research findings; and (3) facilitating the integration of research into policymaking and program management processes. Following a thorough review process, including scientific and technical quality assessment of projects by external reviewers, an independent adjudication committee selected two innovative interventions to be implemented in LMIC settings.

### Implementation research and communities of practice (CoPs)

The first approach (henceforth ‘INSP approach’), coordinated by the National Institute of Public Health in Mexico (Instituto Nacional de Salud Pública, INSP) [25], focused on the use of implementation research (IR), i.e. the scientific study of the implementation of health policies, programmes and interventions in diverse real world settings and within the existing range of health systems [26]. IR can address or explore any aspect of implementation, including the contextual factors affecting implementation, the processes of implementation themselves, and the outcomes, or end-products, of the implementation under study [26]. The importance of IR has been recognized in scaling up maternal and reproductive health program interventions and strengthening the health systems as a whole [27]. However, in the same way as other countries in the Mesoamerican region, Mexico and Nicaragua have exhibited low capacity to undertake, support and use public health research, including IR applied to maternal health [28]. The INSP approach aimed to enhance research utilization by maternal health program management teams and implementers in selected regions of Mexico and Nicaragua. The initiative focused on building the capacity to demand, access and use evidence, while facilitating the integration of research into routine management processes. The approach was implemented in three states of Mexico (Hidalgo, Morelos, Veracruz) and three departments in Nicaragua (Chontales, Jinotega, Matagalpa), from May 2013 to March 2015.

In the intervention states and departments (n = 221 participants), the importance of strengthening interactions between researchers and health personnel at state and community levels was emphasized, and the approach promoted a participatory IR method through the establishment and training of Communities of Practice (CoPs) [29]. CoPs are formed by people who engage in a process of collective learning in a shared domain of human endeavour, i.e. “*groups of people who share a concern or a passion for something they do and learn how to do it better as they interact regularly*” [30]. In Mexico and Nicaragua, the CoPs included researchers, healthcare professionals and health system stakeholders.

**Fig. 1:**
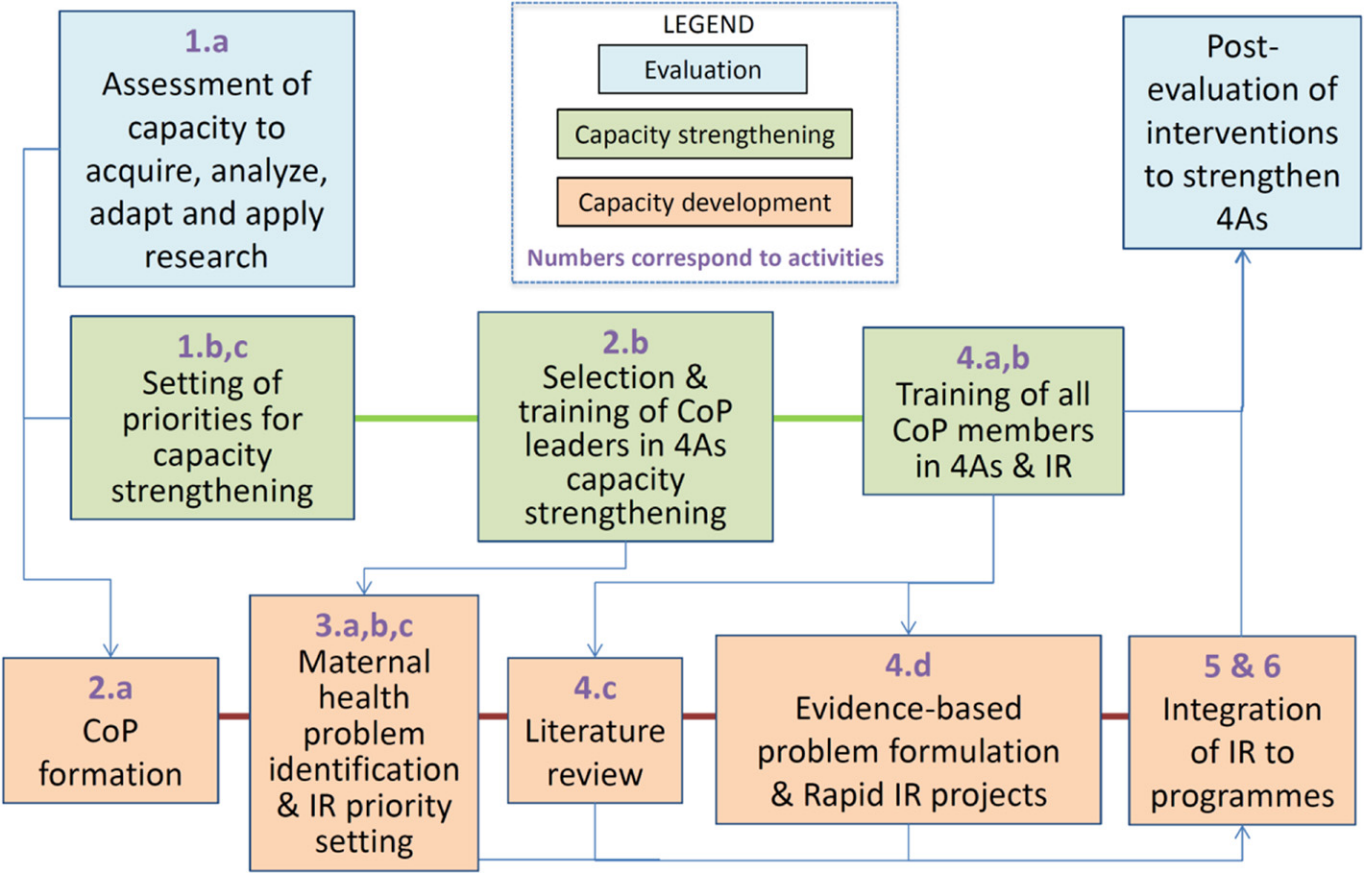
The National Institute of Public Health of Mexico (INSP) approach

**Fig. 2:**
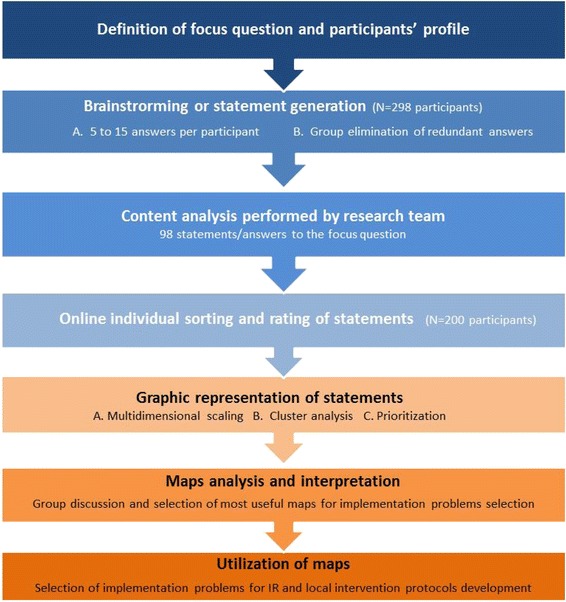
Systematization of tacit knowledge process. Source: Modified from [32]

The INSP approach started with an assessment of the maternal health programme staff’s abilities to acquire, analyse, adapt and apply (4As) research results, based on an adaptation of a self-assessment tool from the Canadian Foundation for Healthcare Improvement – formerly Canadian Health Services Research Foundation (Additional file 1) [31]. The adapted 4As tool was pilot tested with maternal healthcare professionals to assess their understanding of the concepts (n = 10) and to measure internal consistency, using the Cronbach Alpha test (n = 90). The tool showed high consistency for abilities to acquire (α = 0.88), analyse (α = 0.80), adapt (α = 0.92) and apply (α = 0.94) evidence. The results of the 4As assessment helped identify the needs in capacity strengthening towards greater use of evidence in maternal health programmes. Gaps in 4As capacities were addressed by training the CoPs in priority setting for IR, literature review and evidence-based problem formulation (Fig. 1 illustrates how the INSP approach was implemented). In order to identify and systematize tacit knowledge on implementation problems and research priorities, the INSP approach employed concept mapping, allowing systematization of stakeholders’ perceptions, in addition to problems and priorities identified by research processes. Concept mapping allowed the ideas of a group of people about a certain subject to be organized and graphically presented [32], with the aim of extracting and systematizing the tacit knowledge of CoPs members about the implementation challenges in the field of maternal health (Fig. 2). The concept mapping started with a workshop to define the focus question of the exercise, namely: “According to your experience, what are the health system problems that constitute an obstacle for the maternal health program you are working in to achieve its goals?” The CoP members were then organized in small groups to write answers to the focus question, and the research team further collated and synthesized answers from all CoPs, eliminating duplicates across groups and reducing the list to 98 statements.

In the second workshop, participants used an online platform to sort the 98 statements into 5–20 conceptual clusters according to content. They rated each statement using a Likert scale, according to its importance (1 = not important, 5 = of vital importance) and feasibility of solving each problem/statement (1 = cannot be solved, 5 = already being solved). The research team reviewed each member’s information and generated clusters using multidimensional scaling and a correlation matrix, then prioritized clusters using importance and feasibility ratings. Researchers and CoPs collaboratively designed and selected useful cluster maps to form the basis of discussions informing the selection of implementation issues to be addressed. The average values of importance and feasibility ratings were also used to generate correlations at cluster and statement level. Considering the highest scores for importance and feasibility for each problem, CoPs selected specific problems to be addressed through IR projects and local intervention protocols.

The IR proposals were then produced within each CoP, with technical support by INSP on fieldwork data processing and interpretation. The workshops and technical support were provided either through online training platforms or on-site interactions. The maternal health IR findings and literature reviews were then discussed, based on which CoPs developed decision tools that would facilitate the integration of research into program implementation. Finally, pilot projects to integrate these decision tools were suggested to policymakers (Fig. 1).

Throughout the study, we carried out a continuous process evaluation of the CoPs and we conducted a survey of participants to assess the influence of the INSP approach in supporting maternal health programme implementation (Additional file 2). All participants gave informed consent to partake in concept mapping and CoPs, and ethical approval for the initiative was obtained from the Research Ethics Committee of the National Institute of Public Health in Mexico and the Centro de Investigaciones y Estudios de la Salud de la Universidad Nacional Autónoma de Nicaragua.

### Policy BUDDIES

The second approach, called the Policy BUilding Demand for evidence in Decision making through Interaction and Enhancing Skills (Policy BUDDIES) initiative, was established and led by the Centre for Evidence-based Health Care [33], Faculty of Medicine and Health Sciences, at Stellenbosch University in South Africa. The objective of the Policy BUDDIES initiative was to enhance the capacity of sub-national policymakers involved in programs related to Millennium Development Goals 4, 5 and 6 to ask for, demand and use systematic review evidence to inform policymaking in South Africa and Cameroon. The project promoted policymakers’ greater uptake of findings from systematic reviews, by particularly focusing on insufficient communications between researchers and policymakers as the main barrier to evidence uptake [34]. In addressing this issue, principal investigators considered a process of evidence-informed policymaking from three aspects: producer-push (research production), user-pull (demand for evidence) and exchange (deliberate dialogues between researchers and policymakers) [35]. The objective of the approach focused on encouraging exchange of dialogues between researchers and policymakers through a buddying process. Through this process, an evidence-based health field expert or KT expert (the ‘buddy’) was linked to health programme managers and programme coordinators, especially women, at sub-national level.

The building blocks of the buddying process were a baseline situational analysis to identify enablers and barriers in the environment and existing networks, complemented by capacity building workshops tailored to the needs of policymakers, in order to engage them in identifying a number of priority questions [36]. The issues were then shortlisted, prioritised, and discussed with the policymakers in subsequent meetings and dialogues, representing the backbone of the buddying process.

**Fig. 3:**
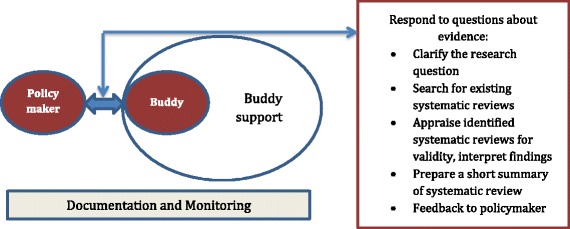
The Policy BUDDIES approach

The interactions between buddies and policymakers entailed specific roles of both parties to encourage both producer-push and user-pull efforts. Firstly, the buddies provided support to refine questions and search for existing systematic reviews and summary of reviews (e.g. SUPPORT summaries [37], existing policy briefs, and related resources). Through regular communication with the designated policymaker, buddies also enhanced their own understanding of the policymakers’ environment and research needs, and assisted them in integrating evidence into policymaking. In turn, policymakers engaged with the buddies to identify and prioritize research questions towards evidence uptake into policy development and implementation. Each priority question or topic was documented as a case study to be a component of the overall buddying process. The buddying approach focused on exchanges between policymakers and buddies, while providing assistance to buddies through a support network (Fig. 3 illustrates how the buddying approach was implemented).

A mixed-methods, realist evaluation of the Policy BUDDIES initiative was carried out by PATH in May 2015, which included the review of policy and project documents (i.e. project proposals, project technical reports, the project workshop report, project meeting minutes and emails, messages between researchers on an online forum, policy documents, technical/evidence inputs from the researcher buddies, and news media), in-depth interviews with policymakers and project staff, and a focus group discussion with researcher buddies [38]. All qualitative data collection was performed by an independent evaluator who used open-ended lines of questioning based on pre-determined topic guides around themes related to the policy case, the engagement and relationship between the policymaker and buddy, and the use of evidence at both individual and organizational levels for the particular policy case. Interviews were audio recorded and notes were taken by a note-taker. Following coding, thematic content analysis was performed. All participants gave informed consent to partake in the evaluation. Ethical approval for the Policy BUDDIES initiative was obtained from the Stellenbosch University Health Research Ethics Committee and the National Ethics Committee for Research on Human Subjects in the Ministry of Public Health in Cameroon. We further undertook a document analysis of the evaluation report to draw key lessons learnt on the process and impact of the Policy BUDDIES approach.

## Results and Discussion

The INSP and Policy BUDDIES approaches put forth a collaborative understanding of complex evidence-informed policymaking processes. The initiatives implemented in Mexico, Nicaragua, South Africa and Cameroon provided insights on practical strategies going beyond traditional KT models, towards processes that actively engaged policymakers and researchers as equal peers. Detailed results from the studies are reported elsewhere [36].

In Mexico, following the strong ownership of IR findings facilitated by CoPs, key local health organizations started to utilize the evaluation forms to periodically monitor the quality of care provided by maternal health programs. In the Mexican state of Morelos, the IR results identified by the CoP shed light on the failures in the training workshops to detect warning signs in pregnant women, leading to a re-organisation of activities by the Secretary of Health. By focusing on strengthening the group structure, the CoPs also addressed problems stemming from the frequent rotation of experienced personnel at state and local levels. In Mexico, health programme personnel reported that the INSP approach “*contributed to improve their capacities to identify and use evidence in solving implementation problems*”.

In Nicaragua, while a couple of sanitary emergencies were an obstacle for the timely achievement of specific goals, the structure of the CoPs assisted the management and control of the outbreaks of chikungunya and dengue. This experience gave an interesting lesson on the utility of this innovative organization of healthcare professionals in enhancing the responsiveness of the health system.

In South Africa, the engagements supported by the Policy BUDDIES project contributed to the policy debate on decentralization of antiretroviral initiation/maintenance (where researcher buddies summarised, presented, and discussed findings from systematic reviews with policymakers) and to the guidelines on prevention of mother-to-child transmission of HIV (where researcher buddies appraised the existing guideline and discussed the appraisal with policymakers). In addition, the buddying process informed a policy framework for medication adherence for chronic diseases, including both HIV and non-communicable diseases. In the evaluation of the buddying experience, policymakers reported that Policy BUDDIES helped them “*recognise the value of research evidence to their daily work*” and ultimately fostered greater demand for policy-relevant knowledge [38]. Beyond catalysing existing requests for research findings, the approach triggered new appetite for science, as policymakers expressed a need for additional evidence on implementation and operational strategies [38].

In Cameroon, the focus was on the implementation of polices, whereas in South Africa, it included both policy development and implementation. The ‘buddying’ aspect of the Policy BUDDIES approach was implemented with high intensity and fidelity in South Africa, while the uptake was slower in Cameroon. At the time of the study, Cameroon was responding to an outbreak of poliomyelitis, which strained the time and resources available to policymakers to engage in the buddying process.

### Level of policymakers

Both the INSP and Policy BUDDIES approaches provided knowledge on evidence uptake processes according to the level of policymakers involved. Seeking insights at the interface between the health system and local communities, the INSP project in Mexico and Nicaragua focused on the doctors, nurses and administrative staff at the frontline of maternal health programmes rather than high-level decision makers. The recognition that tacit knowledge is a valuable source of evidence fostered the empowerment of these frontline staff and had a significant impact when the latter presented the IR findings to high-level policymakers. On the occasion, the new capacities of maternal health personnel were recognised, and the evidence communicated was praised for being closely related to the “real problems” faced in implementing different policies and programmes. The value of IR findings to support and strengthen health systems was also clearly identified by policymakers and CoP leaders. Furthermore, understanding how policymakers used and valued systematized tacit knowledge and the literature review findings empowered the members of the CoPs, who expressed “*a sense of value and satisfaction*” and “*confidence in their capacities to generate pertinent evidence to improve the maternal programs in which they work*”.

In turn, the Policy BUDDIES initiative engaged sub-national policymakers working on chronic non-communicable diseases, nutrition and task shifting for antiretroviral therapy. In South Africa, provincial governments have constitutional obligations to respect laws passed at national level, yet sub-national policymakers can also develop their own context-specific policies within this framework [38]. Provincial policymakers involved in Policy BUDDIES thus benefited from the capacity and agency [39] to plan and implement sub-national health policies. Policymakers reported that “*Policy BUDDIES gave* [them] *more confidence to address the claims of powerful experts in policy discussion*” [38], thus acting as an empowerment mechanism.

In Cameroon, the approach involved sub-national policymakers whose opportunities for evidence-informed decisions were hindered by long planning cycles and the concentration of authority at national level. Capacities of policymakers in finding and interpreting evidence were also identified as an important barrier to evidence-informed policymaking at sub-national level, in addition to lack of institutional support and incentives to use evidence [38]. The difference between South Africa and Cameroon in implementing the Policy BUDDIES intervention can thus be explained by factors pertaining to competing policy priorities and timeliness of policymaking cycles, as well as policymakers’ agency and perception of research evidence.

### Tacit knowledge

Tacit knowledge corresponds to individual knowledge acquired by professionals throughout their everyday practice and experience and is distinct from explicit knowledge (formally expressed and meant to be shared) as it is only occasionally shared in informal ways and contexts [40, 41]. In order to understand the implementers’ tacit knowledge, the INSP approach included a unique feature whereby ideas and opinions generated from brainstorming were refined through a process of crowd-sourcing, to the point at which such opinions became coherent and robust enough to form the basis of policy. In IR, concept mapping is of a particular value in transforming tacit knowledge into explicit and systematically organized knowledge and organizational resource [42]. Responses to the over-arching question on health system failures in maternal health programs addressed a wide range of topics, including drug shortages, human resource restraints, infrastructure, financial and administrative problems, pay and incentives, and the cultural background of women coming into the clinics. The analysis of the concept mapping results, as well the discussion among CoPs, underlined the importance of cultural factors on the implementation of the maternal health programmes, particularly with regard to antenatal and postnatal care. Low utilisation rates were disproportionately observed among women from poor and indigenous communities. On the other hand, the analysis of the concept mapping results highlighted the CoPs’ general perception of quality of care as the main issue that needed to – and could be – addressed from different angles. These findings were also policy-relevant, as they highlighted the need to develop programmes addressing the attitude and role of healthcare practitioners in promoting the use of maternal health services. Concurrently, they provided useful knowledge to support the development of health education and promotion programmes and community-based outreach initiatives to foster timely utilization of care by women.

### Differences, commonalities and lessons learned

As summarized in Table 1, the INSP and Policy BUDDIES approaches differed with regards to the type of research evidence being used for policymaking purposes, the stakeholders involved in the evidence-to-policy processes, the stages in policy formulation and implementation, and the engagement strategy between researchers and policymakers. The two experiences thus emphasize the importance of fit-for-purpose approaches, as different phases and needs in policymaking cycles require adapted policy-relevant mechanisms and products.

**Table 1:** Comparison of key indicators between the INSP and Policy BUDDIES approach

Comparative indicator	Evidence-to-policy approach	
	Communities of practice	Policy BUDDIES
Stakeholders involved	Frontline policy/programme managers and implementers	Sub-national policymakers
Policy cycle	Improving implementation processes	Policy planning, development and adaptation
Type of research evidence	Systematised tacit knowledge and implementation research findings	Systematic reviews and derivative products of evidence syntheses
Engagement process	Workshops and online communities of peers	Dyads and one-on-one relationship building
Key lessons learned	• Engagement of implementers was critical in the conduct of research that responded to identified challenges, and facilitated greater buy-in and uptake of results • Peer learning – through sharing of experiences and challenges – facilitated problem solving • Tacit knowledge important in the formulation of research questions and identification of solutions to implementation barriers • Use of implementation research findings improved when research is embedded within the program process • Complex real-world implementation requires continuous dialogue and engagement • Need to strengthen/support capacities of policy/programme managers and implementers in using implementation research and other forms of knowledge	• Peer support/buddying helped policymakers to value and use research evidence • Capabilities and opportunities for change should be considered when trying to promote research uptake • Complexity and evolving nature of policymaking require iterative exchanges • Collaborations between researchers and policymakers need to be meaningful, equal and built on trust • Need to strengthen/support capacities of policymakers in using evidence syntheses and other forms of knowledge • Researchers need to be sensitized to the policy environment and information needs of policymakers • Institutional support and incentives for using evidence are important barriers/facilitators for policymakers to engage in knowledge generation and use

Both approaches also presented important commonalities in relation to collaborative processes, ownership of policymakers and continuity in the engagement between researchers and policymakers. The initial steps of the two approaches involved a mapping/scoping exercise as a means to assess the needs of policymakers. A strong participatory approach was also put forth in all settings, so as to foster equal partnerships and build a culture of trust among stakeholders. In addition, both approaches involved processes to ensure the robustness and validity of the evidence being used, including but not limited to concept mapping and assessment of scientific quality, as well as contextualisation of research findings. Due consideration was also provided to the relevance and adequacy of knowledge used in support of specific policy needs. Although the collaborative approaches were implemented in different settings and health systems across sub-Saharan Africa and Latin America, these similarities enabled the following cross-cutting lessons from common experiences at the interface between policy and research environments.

### Empowerment of policymakers

In South Africa, policymakers mentioned that researcher buddies were perceived to be more neutral and objective than other internal and external academic and clinical experts involved in policymaking with a “*perceived bias towards their findings*” [38]. The policymaking process was reported to be influenced by group dynamics where “*a few big opinion formers, mostly professors, have their own research agendas*” [38]. A similar balance of power was experienced by frontline implementers in Mexico, who used the IR findings fostered by the CoPs to engage with high-level policymakers. One of the more compelling aspects of IR is its capacity to engage frontline practitioners, drawing on their tacit knowledge and perceptions to inform policy design [26]. The policymakers interviewed across settings underlined that inter-linkages with researchers empowered them to convince other policy stakeholders of the importance of using research evidence. Close collaboration between researchers and health system staff through practical two-way learning processes – for instance, the experience of integrating CoPs and including tacit knowledge as relevant evidence – also gave ways to new approaches for horizontal exchanges. This type of collaborative relationship fostered the perception among health officials that research is not externally imposed, as it informed their understanding of real world implementation challenges.

### Iterative collaboration

The positive outcomes identified in both the CoP and buddying approaches showed that the collaboration and iterative exchanges seemed better aligned to the evolving nature of policy planning and implementation. Traditional knowledge dissemination activities are mostly conceived as stand-alone endeavours, while complex and changing policymaking environments are more tailored to numerous interactions between research and policy stakeholders. The initiatives also made use of information and communication technologies to catalyse exchanges and collaborations among stakeholders, namely an online sharing platform (EZcollab) in South Africa and social media platforms in Mexico and Nicaragua. The use of information and communication technologies in intervention settings supported the synchronicity of policymakers’ demands with input provided by researchers. Such lessons learned about the usefulness of e-health technologies are crucial, as timely access to relevant research findings was reported as a compelling determinant of the use of evidence in health policymaking [19].

### Leadership development

The two pilot projects showed the crucial role of health systems champions and leaders, highlighting the importance of leadership development for both producers and users of research evidence to strengthen LMIC health systems. Motivated champions were key drivers of the ownership of policymakers and the engagement of parties in both the CoP and buddying approaches, and they played a pre-eminent role in stressing the value of science in policymaking. Hence, our findings are consistent with previous evidence underlining that engaged leadership is paramount to steward complex and pluralistic health systems [43].

In addition to strengthening individual capacities in the policy arena, the two multi-site projects also highlight the importance of developing the researchers’ understanding of policy environments and their mindfulness of policymakers’ needs [36]. Strengthening the researchers’ abilities to involve policymakers can, in turn, build their own influence and leadership towards greater policy engagement in the research community.

### Institutional capacity strengthening

Both approaches showed that pathways through which research enters into policy are mediated by institutional arrangements that influence the interactions between policymakers and producers of research [44]. In South Africa, while guidance on policy development exists, it does not encompass the explicit use of research evidence [38]. Furthermore, the need identified by sub-national policymakers in South Africa to exchange learning-by-doing experiences suggests the importance for interventions and structured processes to share tacit knowledge across settings and programmatic areas. Consequently, successful efforts to foster evidence uptake require attention to organizational settings and procedures as well as incentives, governance and enabling environments [45].

## Conclusions

The evidence-informed policymaking approaches implemented in South Africa, Cameroon, Mexico and Nicaragua highlight the importance of cross-learning to integrate research into complex health systems policymaking. While the interventions were different in design, processes and, to some extent, target audiences and types of research evidence used, the commonalities in the multi-site experiences support cross-cutting lessons and conclusions, including the importance of trust- and relationship-building. Both approaches underline the need to develop an equal and continuous collaboration between policymakers and researchers in order to foster mutual understanding and benefits. In turn, such exchanges strengthen the capacities of policymakers to demand, appraise and use research evidence, while developing the researchers’ knowledge of policy realities and their ability to provide timely, appropriate support. In harnessing ongoing exchanges and collaborations between stakeholders, both the buddying and CoP approaches also challenge the vision of policymakers as passive recipients of ‘translated’ or synthesised research.

The examined approaches stress the importance of developing fit-for-purpose interventions tailored to specific contexts and policy needs. In designing and implementing collaborative approaches to foster evidence uptake, due consideration should be provided to the type of evidence demanded by policymakers, the targeted steps in policymaking cycles, and the stakeholders’ competencies towards evidence-informed policymaking. In addition, this work highlights the need for an a priori mapping/scoping exercise, to understand the agency of the stakeholders in countering policy resistance and bringing about real changes in health policies and programmes. As such, interventions aiming to foster the use of research evidence should firstly engage in a thorough situational analysis to assess the capacities of policymakers to demand, appraise and use evidence in order to adopt and implement appropriate evidence-to-policy measures. The multi-site experiences also highlight the necessity to develop the capacities of both policymakers and researchers to engage on a continuous and timely basis.

Although supporting evidence-informed policy by fostering peer-to-peer exchanges seems necessary, it also appears to be insufficient to continuously improve health policies and programmes. The two evidence-to-policy approaches suggest that adopting institutional rules and developing a supportive governance culture help incentivize evidence uptake. Due consideration should be provided to organisational and institutional arrangements to support evidence-informed policymaking, providing space to collaborative approaches such as buddying and CoPs. Health systems should also provide stronger incentives for dialogues between policymakers and researchers through formalised processes and enabling structures and environments. Formalised processes should include explicit incentives to demand and use evidence, as well as time and space for inter-linkages between policymakers.

Research uptake does not follow a linear and deterministic pathway towards evidence-informed policy. Interventions implemented in real world policy-settings should be flexible so as to adapt to heterogeneous contexts and complex realities. Furthermore, approaches fostering the use of evidence should be embedded in continuous implementation cycles in order to account for the evolution of policymaking processes. Embedding the use of research would thus be more tailored to the complex adaptive nature of health systems, as put forth by the systems thinking literature applied to health systems strengthening [46–48]. Finally, policies should be informed by a broad knowledge base, from scientific and grey literature to tacit experience through locally contextualised evidence. A comprehensive conception of knowledge will, in turn, favour its relevance and application to complex decisions improving the performance and responsiveness of health systems and, subsequently, better health services delivery.

## Additional files

Additional file 1. The adapted 4As self-assessment tool. (PDF 441 kb) Additional file 2. Survey questionnaire for participants in the INSP intervention. (PDF 309 kb)

## Abbreviations

4As: Acquire, assess, analyse and adapt research evidence
INSP: Instituto Nacional de Salud Pública (National Institute of Public Health, Mexico)
IR: Implementation research
KT: Knowledge translation
Policy BUDDIES: Policy BUilding Demand for evidence in Decision making through Interaction and Enhancing Skills
CoP: Community of practice
LMIC: Low- and middle-income country

## References

[1] El-Jardali F, Lavis J, Moat K, Pantoja T, Ataya N (2014). Capturing lessons learned from evidence-to-policy initiatives through structured reflection. Health Res Policy Syst..

[2] Cordero C, Delino R, Jeyaseelan L, Lansang MA, Lozano JM, Kumar S (2008). Funding agencies in low- and middle-income countries: support for knowledge translation. Bull World Health Organ.

[3] Syed SB, Hyder AA, Bloom G, Sundaram S, Bhuiya A, Zhenzhong Z, Kanjilal B (2008). Future Health Systems: Innovation for Equity. Exploring evidence-policy linkages in health research plans: a case study from six countries. Health Res Policy Syst.

[4] Daire J, Gilson L, Cleary S. Developing leadership and management competencies in low and middle-income country health systems: a review of the literature. 2014. http://resyst.lshtm.ac.uk/sites/resyst.lshtm.ac.uk/files/docs/reseources/WP4_Developing%20leadership%20and%20management%20competencies.pdf. Accessed 21/01/2015.

[5] de Savigny D, Adam T (2009). Systems thinking for health systems strengthening.

[6] Smits PA, Denis JL (2014). How research funding agencies support science integration into policy and practice: an international overview. Implement Sci.

[7] Mitton C, Adair CE, McKenzie E, Patten SB, Waye Perry B (2007). Knowledge transfer and exchange: review and synthesis of the literature. Milbank Q.

[8] Lavis JN, Boyko JA, Oxman AD, Lewin S, Fretheim A (2009). SUPPORT Tools for evidence-informed health Policymaking (STP) 14: Organising and using policy dialogues to support evidence-informed policymaking. Health Res Policy Syst..

[9] Gagliardi AR, Webster F, Perrier L, Bell M, Straus S (2014). Exploring mentorship as a strategy to build capacity for knowledge translation research and practice: a scoping systematic review. Implement Sci..

[10] Ward V, Hamer S, House A (2009). Knowledge brokering: the missing link in the evidence to action chain?. Evid Policy.

[11] Puljak L. Using social media for knowledge translation, promotion of evidence-based medicine and high-quality information on health. J Evid Based Med. 2015. Ahead of print. 10.1111/jebm.12175 26372327

[12] Murphy K, Fafard P (2012). Taking power, politics, and policy problems seriously: the limits of knowledge translation for urban health research. J Urban Health.

[13] Greenhalgh T, Wieringa S (2011). Is it time to drop the 'knowledge translation' metaphor? A critical literature review. J R Soc Med.

[14] Orem JN, Mafigiri DK, Marchal B, Ssengooba F, Macq J, Criel B (2012). Research, evidence and policymaking: the perspectives of policy actors on improving uptake of evidence in health policy development and implementation in Uganda. BMC Public Health..

[15] Davison CM, Ndumbe-Eyoh S, Clement C (2015). Critical examination of knowledge to action models and implications for promoting health equity. Int J Equity Health.

[16] Lavis JN, Lomas J, Hamid M, Sewankambo NK (2006). Assessing country-level efforts to link research to action. Bull World Health Organ.

[17] World Health Assembly. Resolution on health research. 2005. http://apps.who.int/gb/ebwha/pdf_files/WHA58-REC1/english/A58_2005_REC1-en.pdf.

[18] Lavis J, Davies H, Oxman A, Denis JL, Golden-Biddle K, Ferlie E (2005). Towards systematic reviews that inform health care management and policy-making. J Health Serv Res Policy..

[19] Oliver K, Innvar S, Lorenc T, Woodman J, Thomas J (2014). A systematic review of barriers to and facilitators of the use of evidence by policymakers. BMC Health Serv Res..

[20] Oliver K, Lorenc T, Innvaer S (2014). New directions in evidence-based policy research: a critical analysis of the literature. Health Res Policy Syst..

[21] World Health Organization. WHO Strategy on Health Policy and Systems Research: Changing Mindsets. 2012. http://www.who.int/alliance-hpsr/alliancehpsr_changingmindsets_strategyhpsr.pdf. Accessed 25/02/2015.

[22] Aarons GA, Hurlburt M, Horwitz SM (2011). Advancing a conceptual model of evidence-based practice implementation in public service sectors. Adm Policy Ment Health.

[23] Bigdeli M, Javadi D, Hoebert J, Laing R, Ranson K (2013). Alliance for Health Policy and Systems Research Network of Researchers on Access to Medicines. Health policy and systems research in access to medicines: a prioritized agenda for low- and middle-income countries. Health Res Policy Syst.

[24] Ranson K, Law TJ, Bennett S (2010). Establishing health systems financing research priorities in developing countries using a participatory methodology. Soc Sci Med.

[25] Instituto Nacional de Salud Pública (INSP). Overview of the National Institute of Public Health in Mexico. 2015. http://www.insp.mx. Accessed 25/01/2015.

[26] Peters D, Tran N, Adams T (2013). Implementation Research in Health: A Practical Guide.

[27] Kengeya-Kayondo J, Gonzalez Block MA, Bochorisvili I (2011). Implementation Research for the Control of Diseases of Poverty: Strengthening the Evidence Base for Access to New and Improved Tools, Strategies and Interventions.

[28] Gonzalez Block MA, Gonzalez Robledo LM, Cuadra Hernandez SM (2013). Diagnosis of capacity to perform essential public health functions in the Central American countries, the Dominican Republic, and the Mexican states of Chiapas and Quintana Roo. Rev Panam Salud Publica.

[29] Minkler M, Salvatore A, Colditz G, Brownson RC, Proctor EK (2012). Participatory approaches for the study design and analysis of dissemination and implementation research. Dissemination and Implementation Research in Health: Translating Science to Practice.

[30] Wenger-Trayner E, Fenton-O'Creevy M, Hutchinson S, Kubiak C, Wenger-Trayner B (2015). Learning in Landscapes of Practice. Boundaries, identity, and knowledgeability in practice-based learning.

[31] CFHI. 4As Model: Acquire, Assess, Analyse and Adapt Evidence. 2016. http://www.cfhi-fcass.ca/Home.aspx. Accessed 15/02/2016.

[32] Trochim W (1989). An introduction to concept mapping for planning and evaluation. Eval Program Plan..

[33] Centre for Evidence-based Health Care. Overview of the Centre for Evidence-based Health Care, Faculty of Medicine and Health Sciences, Stellenbosch University. 2015. www.sun.ac.za/cebhc. Accessed 20/11/2015.

[34] Innvaer S, Vist G, Trommald M, Oxman A (2002). Health policy-makers’ perceptions of their use of evidence: a systematic review. J Health Serv Res Policy.

[35] Lavis J, Ross S, McLeod C, Gildiner A (2003). Measuring the impact of health research. J Health Serv Res Policy.

[36] Naude CE, Zani B, Ongolo-Zogo P, Wiysonge CS, Dudley L, Kredo T (2015). Research evidence and policy: qualitative study in selected provinces in South Africa and Cameroon. Implement Sci..

[37] Lavis JN, Oxman AD, Lewin S, Fretheim A (2009). SUPPORT Tools for evidence-informed health Policymaking (STP). Health Res Policy Syst..

[38] Shearer J, Rupert T. Policy BUDDIES: BUilding Demand for evidence in Decision making through Interaction and Enhancing Skills: Evaluation Report. Seattle: PATH; 2015.

[39] Ritzer G (2000). Modern Sociological Theory.

[40] Nonaka I (1994). A dynamic theory of organizational knowledge creation. Organ Sci.

[41] Kothari AR, Bickford JJ, Edwards N, Dobbins MJ, Meyer M (2011). Uncovering tacit knowledge: a pilot study to broaden the concept of knowledge in knowledge translation. BMC Health Serv Res..

[42] Gonzalez-Block MA, Rouvier M, Becerril V, Sesia P (2011). Mapping of health system functions to strengthen priority programs. The case of maternal health in Mexico. BMC Public Health..

[43] World Health Organization. The World Health Report 2008 – Primary Health Care (Now More Than Ever). 2008. http://www.who.int/whr/2008/en/. Accessed 27/05/2015.

[44] Koon AD, Nambiar D, Rao KD (2012). Embedding of Research into Decision-Making Processes.

[45] OECD (2006). The Challenge of Capacity Development: Working Towards Good Practice.

[46] Plsek PE, Greenhalgh T (2001). Complexity science: the challenge of complexity in health care. BMJ.

[47] Adam T (2014). Advancing the application of systems thinking in health. Health Res Policy Syst..

[48] Peters DH (2014). The application of systems thinking in health: why use systems thinking?. Health Res Policy Syst..

