# Supplementary material for: Enhancing evidence informed policymaking in complex health systems: lessons from multi-site collaborative approaches
Source: Health Res Policy Syst. 2016 Mar 17;14:20. doi: 10.1186/s12961-016-0089-0 (PMC4794922; doi:10.1186/s12961-016-0089-0)
Supplement: Additional file 1. — The adapted 4As self-assessment tool. (PDF 441 kb) [file 12961_2016_89_MOESM1_ESM.pdf]

Fortalecimiento de capacidades para la utilización de investigación de implementación en programas de salud materna

CUESTIONARIO DE AUTO-DIAGNÓSTICO Y GUÍA DE DISCUSIÓN

¿Cómo aprovechar la investigación y la información operativa para mejorar los programas y servicios de salud materna?

Cuestionario Individual

Para mayores informes, ponerse en contacto
con la coordinación del proyecto en su país

México

Dra. Jacqueline Alcalde (jacqueline.alcalde@insp.mx) / Mtro. Víctor Becerril (victor.becerril@insp.mx)

Centro de Investigación en Sistemas de Salud / Instituto Nacional de Salud Pública

Nicaragua

Dr. Miguel Ángel Orozco / Dra. Marcia Ibarra (mibarra@cies.edu.ni)

Centro para Investigación y Estudios en Salud / Universidad Nacional Autónoma de Nicaragua

Marzo 2014

CUESTIONARIO INDIVIDUAL DE AUTO-DIAGNÓSTICO

Antecedentes

El cuestionario original de autodiagnóstico fue diseñado por la Fundación Canadiense para la Investigación en Servicios de Salud, actualmente Canadian Foundation for Healthcare Improvement. Inicialmente fue traducido y adaptado en México por el proyecto Investigación para el Desarrollo de Sistemas Estatales de Salud (INDESES) a partir de la cual se han realizado adecuaciones para ser utilizada para el proyecto de ***Fortalecimiento de capacidades para la utilización de investigación de implementación en programas de salud materna*** **en México y Nicaragua.**

¿Qué es y qué le ofrece este cuestionario de auto-diagnóstico?

Este cuestionario permitirá conocer a usted y sus colaboradores la forma como se obtiene y utiliza la información **operativa y procedente de la investigación en los programas de salud materna**. La finalidad es identificar áreas de oportunidad para contribuir a mejorar procesos y resultados con el uso de evidencia procedente la investigación o la información operativa.

Instrucciones y sugerencias para llenar el cuestionario

- Cada participante recibirá un ejemplar del cuestionario individual.
- Dentro del cuestionario se habla del "**programa**", se refiere al **"programa de salud materna"**
- El cuestionario individual comprende dos secciones:

  I. Datos generales del participante
  II. Diagnóstico: incluye cuatro grupos de preguntas para explorar las capacidades de *A*dquisición, *A*nálisis, *A*daptación y *A*plicación de evidencias científicas y operativas en salud materna

- Cada participante debe leer detenidamente las preguntas y seleccionar **sólo una respuesta (a**quella que considere más adecuada al ámbito donde trabaja).
- Los participantes deben responder todas las preguntas.
- El tiempo de aplicación del cuestionario es de 30 a 40 minutos.
- Al terminar la guía individual, el participante debe integrarse a un equipo para responder la guía de preguntas grupales.

I. Datos generales del participante

1. Estado o Departamento: ____
2. Organización, institución: ____
3. Departamento / unidad / programa: _______________________________________
4. Domicilio:____
5. Fecha de aplicación del cuestionario:_______/______________/_____________
6. Edad __________años
7. Sexo M F
8.

Grado de instrucción		
	1	Primaria incompleta
	2	Primaria completa
	3	Secundaria incompleta
	4	Secundaria completa
	5	Preparatoria incompleta
	6	Preparatoria completa
	7	Licenciatura incompleta
	8	Licenciatura completa
	9	Maestría incompleta
	10	Maestría completa
	11	Doctorado incompleto
	12	Doctorado completo

9. Profesión ____
10. Puesto o cargo actual :____
11. Tiempo de experiencia en el puesto actual: [ ] años y [ ] meses
12. Capacitación que ha recibido en los últimos dos años

Tema de capacitación	Duración	Institución que capacitó

II. Diagnóstico

A1. Adquisición

A1.1 Adquisición de información científica producto de investigación

¿Somos capaces de adquirir resultados de investigación para nuestro programa/institución?

1. Tenemos **personal capacitado** para hacer o buscar **investigación para la toma de decisiones** en el programa/institución
   a) No se tiene personal capacitado para hacer o buscar información de investigación.
   b) Se dispone de al menos una persona con capacidades para hacer o buscar información.
   c) Se dispone de un equipo de personas con capacidades para hacer o buscar investigación, pero no está formado para ello.
   d) Se dispone de un equipo de profesionales con capacidades para hacer o buscar investigación y más de la mitad de ellos tienen maestría. .
   e) Más del 75% del personal tiene capacidades para hacer investigación y cuenta con maestría o doctorado.

2. Nuestro personal dedica **tiempo suficiente** a la investigación para la toma de decisiones.
   a) No dedicamos tiempo a la investigación.
   b) Dedicamos al menos 8 horas de trabajo semanal a investigación.
   c) Dedicamos entre 8 y 12 horas de trabajo semanal a investigación.
   d) Dedicamos entre 12 y 20 horas de trabajo semanal a investigación.
   e) Se dedica más de 20 horas de trabajo semanal a investigación.

3. Nuestro personal tiene **incentivos** (cursos y talleres de capacitación, asistencia a congresos, estímulos y/o compensaciones extras, etc.) **para hacer o buscar investigación** para apoyar la toma de decisiones.
   a) No se cuenta con incentivos para hacer o buscar investigación.
   b) Se premia únicamente en caso de que algún investigador gane un premio en otro lado
   c) Algunas veces hemos recibido incentivos para hacer o buscar investigación.
   d) Una o dos veces al año hemos recibido incentivos para hacer o buscar investigación.
   e) Siempre se premia a los investigadores de acuerdo a su productividad.

4. Nuestro personal cuenta con **recursos** (conocimientos, financiamiento, equipo de cómputo, acceso a biblioteca, Internet, etc.) para hacer o buscar investigación para apoyar la toma de decisiones del programa/institución.
   a) No se cuenta con recursos para hacer o buscar investigación.
   b) Se cuenta con algunos recursos para hacer o buscar investigación
      ☐ con acceso limitado ☐ con acceso adecuado
   c) Se cuenta con la mayoría de los recursos para hacer o buscar investigación
      ☐con acceso limitado ☐ con acceso adecuado
   d) Se cuenta con recursos para hacer o buscar investigación, pero su acceso es limitado.
   e) Se cuenta con todos los recursos para hacer o buscar investigación

5. Tenemos **convenios con expertos externos** que investigan, buscan resultados o monitorean avances de investigación para nuestros programas/institución
   a) No tenemos convenios con expertos externo en investigación
   b) Se está en proceso para establecer convenios con expertos externos en investigación
   c) Se tienen convenios con expertos externos en investigación por requerimiento del programa.
   d) Se tienen convenios con expertos externos en investigación por iniciativa del equipo del programa.
   e) Se tienen convenios permanentes con expertos externos nacionales e internacionales en investigación.

A1.2 Adquisición de información operativa del funcionamiento de nuestro programa o institución.

¿Somos capaces de generar o buscar información operativa que apoye la toma de decisiones para el funcionamiento del programa/institución?

6. Tenemos **personal capacitado** para generar o reunir información operativa para el programa/Institución
   a) No tenemos personal capacitado para generar o reunir información operativa.
   b) Contamos con al menos una persona capacitada para generar o reunir información operativa pero no es suficiente.
   c) Contamos con al menos una persona capacitada para generar o reunir información operativa y es suficiente.
   d) Contamos con un equipo que genera o reúne información operativa del programa pero la información está disponible con retraso.
   e) Contamos con un equipo que genera o reúne información operativa del programa y se

dispone de información oportunamente.

7. Nuestro personal dedica **tiempo suficiente** a generar o reunir información operativa de nuestro programa/institución.
   a) No se dedica tiempo para generar o reunir información operativa.
   b) Se dedica menos de 8 horas semanales para generar o reunir información operativa
   c) Se dedica entre 8 a 12 horas semanales para generar o reunir información operativa
   d) Se dedica entre 12 a 20 horas semanales para generar o reunir información operativa.
   e) Se dedica más de 20 horas semanales para generar o reunir información operativa.

8. Nuestro personal tiene **incentivos** (cursos y talleres de capacitación, asistencia a congresos, estímulos y/o compensaciones extras, etc.) para generar o reunir información operativa del programa/institución.
   a) No se tienen incentivos para generar o reunir información operativa.
   b) Se reciben incentivos para generar o reunir información operativa una vez al año.
   c) Se reciben incentivos para generar o reunir información operativa dos veces al año.
   d) Se reciben incentivos para generar o reunir información operativa más de dos veces al año
   e) Se reciben incentivos para generar o reunir información operativa todas las veces necesarias.

9. Nuestro personal tiene **recursos** (financiamiento, equipo de cómputo, acceso a biblioteca, Internet, etc.) para generar o reunir información operativa de nuestro programa o institución.
   a) No se dispone de ningún recurso para generar o reunir información operativa
   b) Se dispone de pocos recursos para generar o reunir información operativa
   c) Se dispone de la mayoría de ellos para generar o reunir información operativa, pero el acceso es limitado.
   d) Se dispone de la mayoría de ellos para generar o reunir información operativa y el acceso es adecuado.
   e) Se dispone de todos los recursos para generar o reunir información operativa y el acceso es adecuado.

10. Tenemos **convenios con expertos externos** para generar o reunir información operativa de nuestro programa o institución.
    a) No se tienen convenios con expertos externos para generar o reunir información operativa.
    b) Se tienen convenios esporádicos con expertos externos para generar o reunir información operativa, pero son de bajo alcance

c) Se tienen convenios permanentes con expertos externos para generar o reunir información, pero son de bajo alcance
d) Se han establecido convenios permanentes con expertos externos para generar o reunir información, pero son de bajo alcance
e) Se han establecido convenios permanentes para generar o reunir información y son de amplio alcance.

¿Buscamos la información en los lugares adecuados?

11. Buscamos en **revistas científicas** (por ejemplo, por suscripción, Internet, acceso a redes, bases de datos, bibliotecas).
    a) Nunca se busca información en revistas científicas.
    b) Se busca información sólo en revistas en las que tenemos suscripción.
    c) Se busca información en revistas científicas nacionales independientemente de la suscripción.
    d) Se busca información en revistas nacionales e internacionales en las que tenemos suscripción.
    e) Se busca información en revistas nacionales e internacionales independientemente de la suscripción.

12. Buscamos en **boletines o informes** por medio de bibliotecas, acceso a Internet, envío por correo de parte de organizaciones como las instancias departamentales o estatales de salud.
    a) Nunca se busca información en boletines o informes.
    b) Se busca información en boletines o informes nacionales de instituciones con las que tenemos un vínculo directo
    c) Se busca información en boletines o informes nacionales e internacionales de instituciones con las que tenemos algún vínculo.
    d) Se busca información en boletines o informes nacionales y estatales.
    e) Se busca información en boletines o informes nacionales e internacionales independientemente de vinculación con nuestra institución.

13. **Trabajamos con investigadores** a través de reuniones formales e informales.
    a) Nunca trabajamos con investigadores.
    b) Trabajamos con investigadores sólo cuando el programa lo requiere
    c) Trabajamos con investigadores sólo cuando hay una instrucción superior
    d) Trabajamos con investigadores de manera periódica pero su apoyo no está formalizado
    e) Trabajamos con investigadores de manera periódica, su apoyo está formalizado

14. **Aprendemos de nuestros colegas** a través de redes informales o formales para intercambiar ideas, experiencias y las mejores prácticas.
    a) No se cuenta con espacios informales o formales para compartir conocimiento/experiencias con nuestros colegas.
    b) Se comparten conocimientos/experiencias sólo con colegas cercanos.
    c) Se comparten conocimientos y experiencias con colegas cercanos y de otras áreas.
    d) Se comparten conocimientos/experiencias de manera constante con todo el personal.
    f) Se comparten conocimientos y experiencias nacionales e internacionales con todo el personal.

A2. Análisis

A2.1 Análisis de información científica producto de investigación

¿Podemos determinar si la investigación es confiable y de alta calidad?

15. **Tenemos personal con habilidades y herramientas de análisis** para evaluar la **calidad y la confiabilidad** de la investigación.
    a) No se utiliza información procedente de investigación.
    b) Se utiliza información de investigaciones pero no se verifica su confiabilidad.
    c) Se utiliza información de investigaciones y se ha iniciado el proceso de verificación de su confiabilidad.
    d) Se utiliza información procedente de investigación y se verifica su calidad y confiabilidad de manera parcial.
    e) Se utiliza información procedente de investigación y se verificar su calidad y confiabilidad.

16. Tenemos **convenios con expertos externos** capaces de **evaluar la calidad y la confiabilidad** de la investigación que usamos para la toma de decisiones en los programas de salud materna.
    a) No se tiene contacto con expertos externos para evaluar la calidad y confiabilidad de la investigación
    b) Se ha establecido contacto con expertos externos para evaluar la calidad y confiabilidad de la investigación, pero no hay convenio.
    c) Se ha establecido contacto con expertos externos para evaluar la calidad y confiabilidad de la investigación, pero no hay convenio.
    d) La información procedente de la investigación que usamos ha sido evaluada por expertos externos, pero no hay convenio.
    e) La información procedente de la investigación que usamos ha sido evaluada por expertos externos en el marco de un convenio.

¿Podemos determinar si la investigación es pertinente y aplicable a nuestro programa/institución?

17. Tenemos personal capaz de **vincular la investigación** que hacemos o que buscamos **con el programa de salud materna e identifica** similitudes y las diferencias.
    a) No se ha vinculado la investigación con el programa de salud materna.
    b) Se han identificado algunas áreas relevantes para realizar investigación en el programa de salud materna.
    c) Se dispone de protocolos de investigación de áreas relevantes del programa de salud materna.
    d) Se está realizando investigación en áreas relevantes de salud materna.
    e) Se han socializado resultados de investigación que realizó sobre el programa de salud materna.

18. Tenemos **convenios con expertos externos** para **comparar la forma en que opera el programa de salud materna contra estándares producto de la investigación**
    a) No se tiene contacto con expertos externos para hacer esta comparación.
    b) Se ha establecido contacto con algunos expertos externos para hacer esta comparación, pero no se dispone de convenio.
    c) Se tiene apoyo de expertos externos para hacer esta comparación, el convenio está en proceso.
    d) Se tiene apoyo de expertos externos para hacer esta comparación y se tiene convenio firmado.
    e) Se ha elaborado un informe de resultados de esta comparación que se realizó con apoyo de expertos externos, cooperación que está avalada en un convenio.

A2.2 Análisis de información operativa sobre el funcionamiento del programa de salud materna.

¿Podemos determinar si la información operativa del programa de salud materna es confiable y de alta calidad?

19. **Tenemos personal con habilidades y herramientas de análisis** para evaluar la **calidad y la confiabilidad** de la información operativa del programa de salud materna.
    a) No contamos con personal para evaluar la calidad y confiabilidad de la información operativa.
    b) Se ha iniciado la capacitación de uno o más profesionales para evaluar la calidad y confiabilidad de la información operativa.
    c) Se dispone de al menos un profesional capacitado que revisa la calidad y confiabilidad de la información operativa.

d) Se dispone de un equipo de profesionales que revisa la calidad y confiabilidad de la información operativa, pero aún es insuficiente.

e) Se dispone de un equipo de profesionales suficiente para revisar la calidad y confiabilidad de la información operativa.

20. Tenemos **convenios con expertos externos** capaces de **evaluar la calidad y confiabilidad** de la información operativa que utilizamos para la toma de decisiones.

a) No se tiene contacto con expertos externos para evaluar la calidad y confiabilidad de la información operativa

b) Se ha establecido contacto con algunos expertos externos para evaluar la calidad y confiabilidad de la información operativa.

c) Se tiene apoyo de expertos externos para evaluar la calidad y confiabilidad de la información operativa, pero no se tiene convenio.

d) Se ha elaborado un convenio con expertos externos para evaluar la calidad y confiabilidad de la información operativa, pero la colaboración es aún insuficiente.

e) Se dispone de convenios con expertos externos y se han elaborado algunos informes de evaluación de la calidad y confiabilidad de la información operativa.

¿Podemos determinar si la información operativa del programa de salud materna es pertinente y aplicable?

21. Tenemos personal capaz de **utilizar la información operativa** que generamos o reunimos **para evaluar el desempeño del programa de salud materna.**

a) No se cuenta con personal capaz de evaluar el desempeño del programa con la información operativa que se genera.

b) Se cuenta con al menos con una persona capaz de evaluar el desempeño del programa con la información operativa que se genera pero no lo hace

c) Se cuenta con al menos con una persona que evalúa el desempeño del programa con la información operativa que se genera pero lo hace tardíamente.

d) Se cuenta con un equipo de profesionales que evalúa el desempeño del programa al menos dos veces al año.

e) Se cuenta con un equipo de profesionales que evalúa el desempeño del programa, lo realiza dos veces al año y socializa los resultados.

22. Tenemos **convenios con expertos externos que nos apoyan a vincular la información operativa para mejorar el desempeño del programa de salud materna.**

a) No se tiene contacto con expertos externos para vincular la información operativa con el programa.

b) Se ha establecido contacto con algunos expertos externos para vincular la información operativa con el programa.

c) Se tiene apoyo de expertos externos para vincular la información operativa con el

programa y el convenio está en proceso de negociación.

d) Se tiene convenio con expertos externos para vincular la información operativa con el programa pero no la colaboración aún no es suficiente.

e) Se tiene convenio con expertos externos y se han establecido mecanismos de colaboración permanentes.

A3. Adaptación

¿Contamos con personal capaz de sintetizar resultados de investigación y la información operativa de forma comprensible y útil para el programa de salud materna?

23. **Tenemos personal capaz de presentar los resultados de la investigación** de manera **comprensible y útil para el programa/institución.**

a) No se tiene personal capaz de presentar resultados de investigación de manera comprensible y útil.

b) Se tiene al menos una persona capaz de presentar resultados de investigación, pero lo hace de manera poco comprensible.

c) Miembros del equipo de trabajo presentan resultados de investigación que sólo algunos integrantes del equipo la comprenden.

d) Miembros del equipo de trabajo presentan resultados de investigación de manera comprensible

e) Miembros del equipo de trabajo presentan resultados de investigación de manera comprensible y es útil para la toma de decisiones.

24. **Tenemos personal capaz de presentar la información operativa de manera comprensible y útil para nuestro programa o institución.**

a) No se tiene personal capaz de presentar resultados de la información operativa de manera comprensible y útil.

b) Se tiene al menos una persona capaz de presentar resultados de la información operativa, pero lo hace de manera poco comprensible.

c) Miembros del equipo de trabajo presentan resultados de la información operativa que sólo algunos integrantes del equipo la comprenden.

d) Miembros del equipo de trabajo presentan resultados de la información operativa de manera comprensible

e) Miembros del equipo de trabajo presentan resultados de la información operativa de manera comprensible y es útil para la toma de decisiones.

25. **Tenemos personal capaz de vincular resultados de la investigación** con los problemas centrales que enfrentan nuestros **tomadores de decisiones.**
    a) No se tiene personal capaz de vincular resultados de investigación con los problemas que enfrentan los tomadores de decisiones.
    b) El personal responsable de la investigación no conoce los problemas que enfrentan los tomadores de decisiones.
    c) El personal responsable de la investigación conoce los problemas que enfrentan los tomadores de decisiones pero no hay coordinación entre ellos.
    d) El personal responsable de la investigación conoce los problemas que enfrentan los tomadores de decisiones, pero coordinan de manera esporádica.
    e) El personal responsable de la investigación conoce los problemas de salud y apoya de manera permanente a los tomadores de decisiones.

26. **Tenemos personal capaz de informar a los tomadores de decisiones sobre las acciones recomendadas por la investigación.**
    a) No se tiene personal capaz de informar a los tomadores de decisiones sobre las acciones recomendadas por la investigación.
    b) Se tiene personal responsable de investigación pero no tiene capacidad para transmitir a los tomadores de decisiones las acciones recomendadas por la investigación
    c) Se tiene personal responsable de investigación que ocasionalmente transmite a los tomadores de decisión las acciones recomendadas por la investigación.
    d) El personal responsable de investigación transmite de manera clara a los tomadores de decisiones las acciones recomendadas por la investigación.
    e) El personal responsable de investigación apoya a los tomadores de decisión a seleccionar las mejores acciones recomendadas por la investigación.

27. **Tenemos personal capaz de vincular la información operativa** con los problemas centrales que enfrentan nuestros **tomadores de decisiones**.
    a) No se tiene personal capaz de vincular resultados de la información operativa con los problemas que enfrentan los tomadores de decisiones.
    b) El personal responsable de información operativa no conoce los problemas que enfrentan los tomadores de decisiones.
    c) El personal responsable de la información operativa conoce los problemas que enfrentan los tomadores de decisiones pero no hay coordinación entre ellos.
    d) El personal responsable de la información operativa conoce los problemas que enfrentan los tomadores de decisiones, pero coordinan de manera esporádica.
    e) El personal responsable de la información operativa conoce los problemas de salud y apoya de manera permanente a los tomadores de decisiones.

¿Cuenta el programa de salud materna con el apoyo de expertos externos que tienen habilidades para comunicar los resultados de la investigación?

28. **El programa de salud materna tiene acuerdos con expertos externos para presentar resultados de investigación** de manera **comprensible y útil?.**
    a) No tenemos acuerdos con expertos externos para presentar resultados de investigación de manera comprensible y útil.
    b) Esporádicamente establecemos acuerdos con expertos externos que presentan resultados de investigación de manera comprensible y útil.
    c) Tenemos acuerdos con experto externos que presentan la información de manera comprensible y útil solo cuando el programa lo requiere
    d) Tenemos con convenios permanentes con expertos externos pero no siempre ofrecen información comprensible y útil para el programa.
    e) Tenemos convenios permanentes con expertos externos que siempre ofrecen información y recomendaciones útiles para mejorar el programa.

29. **El programa de salud materna tiene acuerdos con expertos externos capaces de vincular los resultados de la investigación** con los problemas centrales que enfrentan nuestros **tomadores de decisiones**.
    a) No se tienen acuerdos con expertos externos para vincular la investigación con los problemas que enfrentan los tomadores de decisiones.
    b) Se tienen acuerdos con expertos externos, pero la vinculación de la investigación con los problemas que enfrentan los tomadores de decisiones no es clara.
    c) Se tienen acuerdos con expertos externos, la vinculación de la investigación con los problemas que enfrentan los tomadores de decisiones es clara y útil pero poco frecuente.
    d) Se tienen acuerdos con expertos externos y la vinculación de la investigación con los problemas que enfrentan los tomadores de decisiones es clara y útil pero poco frecuente.
    e) Se tienen acuerdos con expertos externos y la investigación siempre se vincula con los problemas que enfrentan los tomadores de decisiones.

30. **El programa tiene acuerdos con expertos externos capaces de vincular la información operativa** con los problemas centrales que enfrentan los **tomadores de decisiones**.
    a) No se tienen acuerdos con expertos externos para vincular la investigación con los problemas que enfrentan los tomadores de decisiones.
    b) Se tienen acuerdos con expertos externos, pero la vinculación con los problemas que enfrentan los tomadores de decisiones no es clara.
    c) Se tienen acuerdos con expertos externos, la vinculación con los problemas que enfrentan los tomadores de decisiones es clara y útil pero poco frecuente.
    d) Se tienen acuerdos con expertos externos y la vinculación con los problemas que enfrentan los tomadores de decisiones es clara y útil pero poco frecuente.

e) Se tienen acuerdos con expertos externos y sus resultados siempre se vinculan con claridad con los problemas que enfrentan los tomadores de decisiones.

A4. Aplicación

¿Lideramos con el ejemplo y mostramos cuánto valoramos el uso de la investigación y la información operativa?

31. El **uso de resultados de investigación** es prioritario en el programa de salud materna?
    a) Nunca se usa la investigación en el programa
    b) Se usa la investigación sólo para hacer algunos reportes oficiales.
    c) Se usa la investigación sólo cuando se recibe una instrucción superior.
    d) Se usa la investigación según lo indica la metodología del programa.
    e) Siempre se usa la investigación de manera permanente.

32. Nuestro programa o institución garantiza que el **personal se involucre en las decisiones del programa para el cumplimiento de metas utilizando resultados de investigación.**
    a) El personal nunca se involucra al personal en las decisiones
    b) El personal sólo se involucra en estas decisiones para hacer reportes oficiales.
    c) El personal sólo se involucra en éstas decisiones cuando recibe instrucción superior
    d) El personal se involucra en decisiones si lo indica la metodología del programa.
    e) El personal se involucra en decisiones de manera constante.

33. El **uso de información operativa** es prioritario en nuestro programa o institución.
    a) Nunca se usa información operativa.
    b) Sólo se usa información operativa para hacer algunos reportes oficiales.
    c) Se usa información operativa cuando se recibe una instrucción superior.
    d) Se usa información operativa según lo indica la metodología del programa.
    e) Siempre se usa información operativa, aunque no lo contemple el programa.

34. Nuestro programa o institución garantiza que el **personal se involucre en las decisiones** sobre cómo se vincula la **información operativa** con nuestras metas principales.
    a) El personal nunca se involucra en estas decisiones.
    b) El personal sólo se involucra en estas decisiones para hacer reportes oficiales.
    c) El personal sólo se involucra en estas decisiones cuando se recibe instrucción superior.
    d) El personal se involucra en estas decisiones si lo indica la metodología del programa.
    e) El personal se involucra en estas decisiones de manera constante.

35. La **dirección** del programa/institución **comunica de forma clara las prioridades y la estrategia d**e forma que quienes generan y monitorean la investigación y/o la información operativa conocen lo que se requiere para apoyar las metas.

    a) La dirección del programa nunca comunica las prioridades y estrategia a quienes generan y monitorean la investigación y/o la información operativa.
    b) La dirección del programa comunica las prioridades y estrategia de manera poco clara.
    c) La dirección del programa comunica las prioridades y la estrategia de manera clara pero sólo para definir la postura oficial del gobierno.
    d) La dirección del programa comunica las prioridades y la estrategia de manera clara y constante.
    e) La dirección del programa siempre comunica la estrategia y prioridades de manera clara, constante y útil para negociar intervenciones.

36. **Nos comunicamos internamente** garantizando el intercambio de información en el programa de salud materna

    a) No existe comunicación interna para intercambiar información
    b) Sólo nos comunicamos para intercambiar información con los colegas más cercanos.
    c) Existe comunicación interna esporádica entre áreas.
    d) Se intercambia información para responder ante problemas y cuando está indicado en el programa.
    e) Se intercambia información de manera constante aun cuando no está indicado en la metodología del programa.

37. En nuestro programa o institución **valoramos la flexibilidad, el cambio, y la mejora continua de la calidad** y **recompensamos con recursos** para apoyar estos valores.

    a) No se valora la flexibilidad y el cambio.
    b) Ocasionalmente se aceptan los cambios pero no hay recompensas para apoyarlos.
    c) Ocasionalmente se aceptan cambios y se recompensan estos esfuerzos.
    d) Generalmente se aceptan cambios y se recompensan los esfuerzos
    e) Existe un constante estímulo y recompensa a los cambios.

¿Hay un lugar para aplicar la investigación y la información operativa en el proceso de toma de decisiones en nuestro programa o institución?

38. A la hora de tomar decisiones importantes, normalmente reservamos **suficiente tiempo** para identificar preguntas de investigación y crear, obtener, analizar y considerar los hallazgos de la investigación.
    a) No se dispone de tiempo para identificar preguntas de investigación o sus hallazgos.
    b) Sólo se dedica tiempo a estas actividades cuando se deben elaborar reportes oficiales.
    c) Sólo se dedica tiempo estas actividades cuando se recibe una instrucción superior.
    d) Sólo se dedica tiempo a estas actividades cuando lo exige la metodología del programa.
    e) Se dedica tiempo a estas actividades de manera constante aun cuando no esté estipulado en la metodología del programa.

39. A la hora de tomar decisiones importantes, normalmente reservamos **suficiente tiempo** para revisar información operativa de nuestro programa
    a) No se reserva tiempo para revisar información operativa antes de tomar decisiones.
    b) Sólo revisamos información operativa para elaborar reportes oficiales.
    c) Sólo revisamos información operativa cuando se recibe una instrucción superior.
    d) Sólo revisamos información operativa cuando lo exige la metodología del programa.
    e) Se dedica tiempo a revisar información operativa de manera constante para tomar decisiones acertadas

40. Nuestro equipo directivo tiene **capacidad para evaluar la factibilidad** de diversas soluciones, incluyendo su impacto potencial en el desempeño del programa de salud materna.
    a) El equipo directivo no tiene capacidad para evaluar la factibilidad de diversas soluciones.
    b) El equipo directivo tiene poca capacidad para evaluar la factibilidad de diversas soluciones.
    c) El equipo directivo tiene capacidad para evaluar la factibilidad de diversas soluciones, pero no evalúa el impacto potencial en el desempeño del programa.
    d) El equipo directivo tiene capacidad para evaluar la factibilidad de diversas soluciones y a veces evalúa el impacto potencial en el desempeño del programa.
    e) El equipo directivo tiene capacidad para evaluar la factibilidad de diversas soluciones y su impacto potencial en el desempeño del programa.

41. Los tomadores de decisiones en nuestro programa de salud materna **consideran de manera formal cualquier recomendación** del personal que ha desarrollado o identificado investigación relevante y de alta calidad.
    a) Los tomadores de decisiones del programa no consideran las recomendaciones del personal que realiza investigación.
    b) Los tomadores de decisiones consideran las recomendaciones del personal que realiza investigación solo para incluirla en reportes oficiales.
    c) Los tomadores de decisiones consideran las recomendaciones del personal que realiza investigación sólo cuando hay demanda del nivel superior.
    d) Los tomadores de decisiones consideran las recomendaciones del personal que realiza investigación solo ante un problema que se necesita resolver de inmediato.
    e) Los tomadores de decisiones consideran las recomendaciones del personal que realiza investigación para mejorar los procesos y resultados del programa.

42. Los tomadores de decisiones en nuestro programa o institución normalmente **consideran de manera formal cualquier recomendación** del personal que maneja información operativa relevante.
    a) Los tomadores de decisiones del programa no consideran las recomendaciones del personal que maneja información operativa relevante.
    b) Los tomadores de decisiones consideran las recomendaciones del personal que maneja información operativa relevante.sólo para incluirla en reportes oficiales.
    c) Los tomadores de decisiones consideran las recomendaciones del personal que maneja información operativa relevante.sólo cuando hay demanda del nivel superior.
    d) Los tomadores de decisiones consideran las recomendaciones del personal que maneja información operativa relevante.sólo ante un problema que se necesita resolver de inmediato.
    e) Los tomadores de decisiones sí consideran las recomendaciones del personal que maneja información operativa relevante.para mejorar los procesos y resultados del programa.

43. El personal y los beneficiarios involucrados conocen **cómo y cuándo se toman las decisiones importantes en el programa de salud materna**
    a) El personal y los beneficiarios no saben cómo ni cuándo se toman las decisiones importantes en el programa
    b) El personal y los beneficiarios ocasionalmente conocen cómo y cuándo se toman las decisiones importantes en el programa
    c) El personal y los beneficiarios se enteran de cómo y cuándo se toman las decisiones importantes cuando lo exige la metodología del programa.
    d) El personal y los beneficiarios se enteran de manera regular cómo y cuándo se toman las decisiones importantes ´para el programa.
    e) El personal y los beneficiarios comparten información y se apoyan mutuamente para tomar decisiones importantes para el programa.

44. El personal y los beneficiarios involucrados conocen **cómo y cuándo pueden aportar evidencias científicas o información operativa y cómo serán empleadas en el programa**
    a) El personal y los beneficiarios no conocen cómo ni cuándo pueden aportar evidencias científicas o información operativa para el programa.
    b) El personal y los beneficiarios ocasionalmente saben cómo y cuándo pueden aportar evidencias científicas o información operativa.
    c) El personal y los beneficiarios ocasionalmente saben cómo y cuándo pueden aportar evidencias científicas o información operativa y cómo serán empleadas.
    d) El personal y los beneficiarios saben cómo y cuándo pueden aportar evidencias científicas o información operativa y cómo serán empleadas cuando lo exige la metodología del programa.
    e) El personal y los beneficiarios conocen regularmente cómo y cuándo pueden aportar evidencias científicas o información operativa y cómo serán empleadas.

45. El personal que aporta resultados de investigación y/o información operativa **participa en las discusiones para la toma de decisiones en el programa**
    a) Nunca participa en las discusiones para la toma de decisiones en el programa.
    b) Sólo participa en las discusiones para la toma de decisiones cuando se elaboran reportes oficiales.
    c) Sólo participa en las discusiones para la toma de decisiones cuando se recibe una instrucción superior.
    d) Sólo participa en las discusiones para la toma de decisiones cuando lo exige la metodología del programa.
    e) Siempre participa en las discusiones para la toma de decisiones del programa.

46. Los **investigadores** que forman parte del personal **participan en las discusiones para la toma de decisiones.** (No aplica si no hay investigadores).
    a) Nunca participan en las discusiones para la toma de decisiones del programa.
    b) Sólo participan en discusiones para la toma de decisiones del programa cuando se van a elaborar reportes oficiales.
    c) Sólo participan en discusiones para la toma de decisiones cuando se recibe una instrucción superior.
    d) Sólo participan en discusiones para la toma de decisiones cuando lo exige la metodología del programa.
    e) Siempre participan en discusiones para la toma de decisiones del programa.

47. El personal y los beneficiarios involucrados reciben **retroalimentación** en cuanto a las decisiones tomadas y su racionalidad.
    a) Nunca son retroalimentados sobre las decisiones tomadas.
    b) Sólo ocasionalmente son retroalimentados sobre las decisiones tomadas.
    c) Sólo son retroalimentados sobre las decisiones tomadas cuando lo exige la metodología del programa.
    d) Generalmente son retroalimentados sobre las decisiones tomadas.
    e) Siempre son retroalimentados sobre las decisiones tomadas.

48. **El personal y los beneficiarios** del programa reciben información sobre la influencia de las evidencias científicas y la información operativa disponibles en las opciones del programa o institución.
    a) Nunca reciben esta información.
    b) Sólo reciben esta información cuando se debe evidenciar en los reportes oficiales.
    c) Sólo reciben esta información cuando se recibe una instrucción superior.
    d) Sólo reciben esta información cuando lo exige la metodología del programa.
    e) Siempre reciben esta información.
