# Supplementary material for: Enhancing evidence informed policymaking in complex health systems: lessons from multi-site collaborative approaches
Source: Health Res Policy Syst. 2016 Mar 17;14:20. doi: 10.1186/s12961-016-0089-0 (PMC4794922; doi:10.1186/s12961-016-0089-0)
Supplement: Additional file 2. — Survey questionnaire for participants in the INSP intervention. (PDF 309 kb) [file 12961_2016_89_MOESM2_ESM.pdf]

Fortalecimiento de capacidades para la utilización de investigación de implementación en programas de salud materna

Encuesta para evaluar la utilización de tecnologías de información y comunicación y elementos del proceso general del proyecto

Para mayores informes, ponerse en contacto
con la coordinación del proyecto en su país

México

Dra. Jacqueline Alcalde (jacqueline.alcalde@insp.mx) / Mtro. Víctor Becerril (victor.becerril@insp.mx)

Centro de Investigación en Sistemas de Salud / Instituto Nacional de Salud Pública

Nicaragua

Dr. Miguel Ángel Orozco / Dra. Marcia Ibarra (mibarra@cies.edu.ni)

Centro para Investigación y Estudios en Salud / Universidad Nacional Autónoma de Nicaragua

Enero de 2015

Este instrumento forma parte de la evaluación de procesos. Se debe aplicar al principio y al final del proyecto para detectar los cambios relacionados con el uso de tecnología de la información y la comunicación (TIC) como herramienta esencial para los procesos de comunicación, desarrollo e intercambio en las comunidades de práctica (CoP), tanto en cada estado o departamento como entre los dos países del proyecto.

.

I. Datos generales del participante

1. Estado o Departamento: ____
2. Organización, institución: ____
3. Departamento / unidad / programa: _______________________________________
4. Domicilio:____
5. Fecha de aplicación del cuestionario:_______/______________/_____________
6. Edad [ ] años
7. Sexo [M] [F]
8. Grado de instrucción

1	Primaria incompleta
2	Primaria completa
3	Secundaria incompleta
4	Secundaria completa
5	Preparatoria incompleta
6	Preparatoria completa
7	Licenciatura incompleta
8	Licenciatura completa
9	Maestría incompleta
10	Maestría completa
11	Doctorado incompleto
12	Doctorado completo

9. Profesión ____
10. Puesto o cargo actual :____
11. Tiempo de experiencia en el puesto actual: [ ] años y [ ] meses

12. Capacitación previa que ha recibido en los últimos dos años

Tema de capacitación	Duración	Institución que capacitó

SECCIÓN 1 – TICs

(Esta primera sección del instrumento fue adaptado a partir del elaborado por Nelson Alejandro y Liusmary Ojeda de la Secretaría Académica del INSP)

INSTRUCCIONES

Marque con un círculo la opción que corresponda para cada pregunta, observando que algunas pueden tener más de una respuesta

1. ¿Con qué frecuencia accede a Internet?

a. No accedo a internet (Pasar a la pregunta 7)

b. De vez en cuando (dos o menos veces al mes)

c. Una o dos veces a la semana

d. Todos los días

2. ¿En qué lugar se conecta a Internet con mayor frecuencia? (Puede marcar más de 1 opción)

a. Café internet

b. Desde mi casa

c. Desde el trabajo

d. Otra (menciónela)______________________________

3. **¿Qué tipo de conexión a Internet posee en el lugar en el que se conecta con mayor frecuencia?**
    a. Conexión de banda ancha
    b. Conexión telefónica
    c. No sé
4. **¿Utiliza alguna red inalámbrica (WiFi)?**
    a. Centro comercial
    b. Casa
    c. Trabajo
    d. Universidad/Escuela
    e. Otra, menciónela__________
    f. No me conecto por red inalámbrica
5. **¿Navega en Internet desde su teléfono móvil?**
    a. No
    b. Si
6. **¿Su teléfono móvil soporta conexión a red inalámbrica (WiFi)?**
    a. No
    b. Si
    c. No sé
7. **¿Cuenta con computador en su oficina?**
    a. No
    b. Si

8. ¿Tiene computador portátil?

a. No

b. Si

9. Cuál de los siguientes Buscadores utiliza con mayor frecuencia?

a. Altavista

b. Google

c. Yahoo

d. Otro (menciónelo)________________________

10. ¿Cuál de los siguientes Navegadores utiliza con mayor frecuencia?

a. Internet explorar

b. Mozilla Firefox

c. Netscape Navigator

d. Safari

e. Otro (menciónelo) _______________________

11. ¿Cuántas cuentas de correo electrónico posee?

a. 1

b. 2

c. 3

d. Más de 3

e. No poseo cuenta

12. ¿Utiliza bases de datos virtuales?

a. No

b. Si

13. ¿Utiliza repositorios de información?

a. No

b. Si

14. ¿Posee o publica en algún blog?

a. No

b. Si

15. ¿Seleccione las redes sociales donde posee usuario?

a. Facebook

b. Twitter

c. MySpace

d. Flicker

e. Linked In

f. ¿Otra? Menciónela: _____________

16. ¿Tiene acceso a cámara Web?

a. No

b. Si

c. No sé

17. Ha utilizado alguna plataforma tecnológica como apoyo en sus materias presenciales (Blackboard, Moodle, WebEx, Elluminate)

a. No (Pase a la pregunta 20)

b. Si

*Si su respuesta a la pregunta anterior es **Si** conteste las preguntas 18 y 19.*

18. ¿Qué plataforma ha utilizado? (Puede marcar más de una opción)

a. Blackboard

b. Moodle

c. Webex

19. ¿Considera útil el uso de estas plataformas?

a. No

b. Si

c. No sé

20 ¿Seleccione las herramientas tecnológicas que usa para realizar sus actividades, comunicarse con sus colaboradores y/o trabajar en equipo? (marque todas las que apliquen)

a. Correo electrónico

b. Messenger

c. Skype

d. Teléfono

e. Wikis

f. Google documents

g. Redes sociales

h. Otra (menciónela)____________________

SECCIÓN 2 – Evaluación del proceso

Esta sección del cuestionario tiene como objetivo que los miembros de las comunidades de práctica expresen sus puntos de vista sobre el desarrollo de todo el proyecto a fin de evaluar el proceso. Por favor responda a cada una de las preguntas basándose en su propia experiencia y percepción del trabajo desarrollado.

INSTRUCCIONES

Por favor, escriba su opinión o comentarios personales en los espacios

1. **¿Qué opina usted de la CoP que se conformó para el trabajo en salud materna?**

____

____

____

____

2. **¿Cuáles cree usted que fueron los procesos más importantes desarrollados por la CoP?**

____

____

____

____

3. **¿Qué competencias y habilidades fortaleció usted como integrante la CoP?**

____

____

____

____

4. **¿Considera que aplicó las competencias desarrolladas en su trabajo diario? Por favor, explique cómo.**

____

____

____

____

5. **¿Qué opina de la interacción entre investigadores y personal de los servicios de salud?**

____

____

____

____

6. **¿Considera importante la presencia de un facilitador local? Explique por qué.**

____

____

____

____

7. **¿Cuáles fueron las limitantes más importantes para su participación en la CoP?**

____

____

____

____

8. ¿Qué es lo que más valora de su experiencia de trabajo en la CoP y en el proyecto en general?

____

____

____

____

9. ¿Qué sugiere para mejorar el desempeño de la CoP?

____

____

____

____
